# Non-verbal joint action in healthy adults: a systematic scoping review of EEG-hyperscanning research

**DOI:** 10.1093/scan/nsaf050

**Published:** 2025-05-08

**Authors:** Antonio De Fano, Patrique Fiedler, Filippo Zappasodi, Maurizio Bertollo, Silvia Comani

**Affiliations:** Department of Education and Sports Sciences, Pegaso University, Naples, 80132, Italy; Behavioral Imaging and Neural Dynamics (BIND) Center, University “Gabriele d’Annunzio” of Chieti-Pescara, Chieti, 66100, Italy; Institute of Biomedical Engineering and Informatics, Technische Universität Ilmenau, Ilmenau, 98693, Germany; Behavioral Imaging and Neural Dynamics (BIND) Center, University “Gabriele d’Annunzio” of Chieti-Pescara, Chieti, 66100, Italy; Department of Neuroscience, Imaging and Clinical Sciences, University “Gabriele d’Annunzio” of Chieti–Pescara, Chieti, 66100, Italy; Institute for Advanced Biomedical Technologies (ITAB), University “Gabriele d’Annunzio” of Chieti–Pescara, Chieti, 66100, Italy; Behavioral Imaging and Neural Dynamics (BIND) Center, University “Gabriele d’Annunzio” of Chieti-Pescara, Chieti, 66100, Italy; Department of Medicine and Aging Sciences, University “Gabriele d’Annunzio” of Chieti-Pescara, Chieti, 66100, Italy; Behavioral Imaging and Neural Dynamics (BIND) Center, University “Gabriele d’Annunzio” of Chieti-Pescara, Chieti, 66100, Italy; Department of Neuroscience, Imaging and Clinical Sciences, University “Gabriele d’Annunzio” of Chieti–Pescara, Chieti, 66100, Italy

**Keywords:** joint action, interpersonal interaction, EEG-hyperscanning, interpersonal neural coupling, social neuroscience

## Abstract

Using EEG-hyperscanning, neuroscientists showed that non-verbal joint actions are associated with different patterns of interpersonal neural coupling (INC), influenced by factors such as the type of joint action investigated, the experimental task used, and the analytical approach employed. The present systematic scoping review aims to identify the categories of non-verbal joint actions investigated so far, the experimental tasks and INC measures used, and the related main findings to provide a comprehensive overview of the field. Thirty-nine articles were included in this review. Most studies investigated cooperative and simultaneous joint actions performed with either similar or complementary actions, whereas competitive and turn-based joint actions are under-investigated. Tasks involve interactions mainly based on movement, music, or computer, with movement-based tasks being most relevant to social neuroscience. Several INC approaches were used, with graph theory and phase synchrony being the commonly used. The main findings are organized and discussed according to the analytical approaches used and, for each approach, into groups of joint action categories and tasks. By providing a structured classification of joint action types and highlighting the relationship between tasks and INC methods, this review offers a reference for designing future studies and refining methodological approaches in EEG-hyperscanning research on social interaction.

## Introduction

In everyday life, non-verbal interpersonal interactions, from workplace cooperation to competitive sports, shape social dynamics and decision-making, impacting human health and well-being (Hari et al. 2015). These interactions can involve independent actions aligning towards a common goal, such as driving in a busy road avoiding accidents and reaching the own destination. Alternatively, they can involve interdependent actions requiring mutual coordination to achieve a shared goal that cannot be accomplished individually, like dancing a tango, playing table tennis, or shaking hands. The latter type of interpersonal interaction is often termed joint action (Searle 1990, Clark 1996), where individual intentions (I-intentions) stem from a collective intention (we-intention), making actions mutually dependent (Searle 1990, Bratman 1992, Clark 1996, Sebanz et al. 2006, Newman-Norlund et al. 2007). Although a universally accepted definition of joint action is missing, joint action is commonly described as ‘any form of social interaction whereby two or more individuals coordinate their actions in space and time to bring about a change in the environment’ (Sebanz et al. 2006, p. 70). However, this definition does not clearly differentiate joint action from other forms of interpersonal interactions, such as action imitation.

The study of the neural underpinnings of joint action can lead to significant practical applications not only in social contexts (Oullier and Basso 2010, Krueger and Meyer-Lindenberg 2019), but also in other domains, such as medical (Short et al. 2021, Tucek et al. 2022), educational (Tan et al. 2023) or in work (Astolfi et al. 2015, Zhang et al. 2019, Balconi et al. 2020, Boukarras et al. 2022) or sports environments (Filho et al. 2016, Cassani et al. 2020).

Currently, there are two social neuroscience approaches that play a crucial role in acquiring, understanding, and evaluating new knowledge related to joint action: the multi-person approach (Hari and Kujala 2009, Dumas 2011, Schilbach et al. 2013, Liu and Pelowski 2014) and the real-world approach (Kasai et al. 2015, Matusz et al. 2019, Shamay-Tsoory and Mendelsohn 2019, Hamilton 2021). The first one asserts that the fundamental unit of analysis in joint action experiments must include all involved individuals rather than focusing on each individual separately as in the traditional stand-alone perspective of cognitive neuroscience (Newman-Norlund et al. 2007). The second approach emphasizes that, to understand brain function during joint actions, the experimental settings and paradigms should replicate the complexity, dynamics, and unpredictability typical of real-life situations. This approach places a strong emphasis on the role of body movements as an integral component of natural human behaviour (Kasai et al. 2015, Matusz et al. 2019).

From a methodological standpoint, investigating joint actions using the multi-person and real-word perspectives of social neuroscience involves simultaneously recording data from multiple brains in (inter)-action within ecological environments. This scientific advancement is now permitted by the development of mobile hyperscanning techniques and multi-person data analytical solutions, which together allowed neuroscientists to go beyond the understanding of the within-brain functional mechanisms and explore the between-brain phenomena (Holroyd 2022, Müller 2022). The idea that the brains of two or more interacting individuals may coordinate their activities when engaged in an interpersonal interaction, including joint actions, arises from the intrinsic association between brain and behaviour. In fact, the successful accomplishment of shared goals in joint actions is positively linked to the degree of spatio-temporal behavioural coordination (D’Ausilio et al. 2012, Chang et al. 2017). Therefore, given that brain and behaviour are inherently associated, researchers have hypothesized that also the brains of interacting individuals may dynamically couple their activities to function as a cohesive unit (van der Wel et al. 2021, Holroyd 2022, Müller 2022).

Hyperscanning investigations have been performed using various neuroimaging techniques (Mu et al. 2018, Czeszumski et al. 2020, Nam et al. 2020). However, from the real-world perspective of social neuroscience, electroencephalography (EEG) emerges as the most suitable technique to study the rapid dynamical changes occurring within and between brains of individuals interacting in ecological environments (Czeszumski et al. 2020, Nam et al. 2020, Zamm et al. 2023a). In fact, EEG features high temporal resolution (i.e. < 1 ms; Michel and Brunet 2019; for a between-technique comparison, see Gross 2019) that permits to capture the fast-changing brain dynamics and inter-brain coordination processes occurring during joint actions. Furthermore, the availability of wireless and miniaturized mobile EEG systems allows studies performed in real-world situations and naturalistic conditions that employ tasks also involving gross whole-body movements.

While EEG-hyperscanning has enabled the recording of multiple brains simultaneously, the development of between-brain analyses has permitted to capture the functional brain interconnections occurring between the interacting individuals, a dimension termed interpersonal neural coupling (INC). Currently, there is no consensus on the most suitable data analysis approaches to study INC underling joint action, leading to a diverse set of solutions (Burgess 2013, Zamm et al. 2023b). In this regard, Ayrolles et al. (2021) categorized common INC measures between two or more interacting brains into five categories: (i) phase synchrony measures, which quantify the temporal alignment of the EEG activities; (ii) amplitude/envelope correlation measures, which quantify the linear relationship between the amplitude (or envelope) of the EEG activities shared; (iii) coherence-based measures, which quantify the degree of similarity in the oscillatory activity in specific frequency bands over a given period of time independent from eventual phase shifts; (iv) causality/directionality-based measures, which quantify the causal relationship and/or directionality of information flow within and between brains; (v) other INC measures, including graph theoretical measures that allow to investigate the organization of their functional networks active during the interpersonal interaction.

The current state of the art suggests that joint actions are indeed associated with INC, but with different patterns of coupled EEG activity (Babiloni and Astolfi 2014, Keller et al. 2014, Liu et al. 2018). These differences could be caused by the different analytical approaches used but also due to the specific type of joint action investigated, and to the characteristics of the task employed (Zamm et al. 2023b). In this regard, Liu and Pelowski (2014) emphasized the importance of considering the specific type of interpersonal interaction and the characteristics of the experimental task employed when studying and interpreting the INC results. They proposed a classification system that distinguishes interpersonal interactions in different dichotomous categories: independent and interdependent (based on the task structure); concurrent and turn-based (based on the interaction structure); cooperative and competitive (based on the goal structure).

Despite the growing number of reviews on hyperscanning and social interaction, no study has specifically examined EEG-hyperscanning in the context of joint action. Several recent reviews have explored hyperscanning in various domains, including emotions (De Felice et al. 2025), music (Cheng et al. 2024), teamwork (Réveillé et al. 2024), and methodological aspects of hyperscanning techniques (Hakim et al. 2023, Zamm et al. 2023b). Some reviews have addressed joint action more broadly but without a specific focus on EEG-hyperscanning (van der Wel et al. 2021), while other reviews have examined EEG-hyperscanning in social interactions but without differentiating joint action from other forms of interpersonal coordination (Liu et al. 2018). More importantly, no previous review has explicitly considered the interplay between the nature of the tasks performed during joint action and the analytical approaches employed to interpret the findings. Many studies implicitly assume that interactions between two or more individuals qualify as joint action, without clearly differentiating between joint action and other types of interpersonal interactions. As a result, past reviews have often treated diverse tasks and analytical methods as interchangeable, leading to a conceptual ambiguity. However, joint actions can differ significantly in terms of task characteristics, interaction dynamics, and goal structures, making it essential to analyse them within an appropriate framework. Furthermore, different EEG-based analytical approaches serve distinct scientific purposes, and their application must be carefully contextualized within the specific type of joint action investigated. Given these gaps in the literature, the present systematic scoping review aims to provide a structured synthesis of EEG-hyperscanning research on joint action, addressing the need for a clearer understanding of the relationship between task characteristics and analytical methodologies.

To this aim, we mapped the research field of EEG-hyperscanning on joint action and identified noteworthy trends and critical gaps in the current body of research. We aimed at systematizing the available scientific knowledge, informing future research directions, and fostering a deeper understanding of the brain-to-brain underpinnings of joint actions. Specifically, the current review has two main aims: (i) to identify the experimental tasks as well as the INC measures employed so far, along with the related main findings, and (ii) to organize and discuss these main findings into groups of analytical approaches and, within each approach, into groups of joint action categories and tasks. To achieve these goals, we also proposed a new working definition and classification system of non-verbal joint actions based on Clark’s theoretical framework of verbal joint action.

## Materials and methods

The current systematic scoping review was designed following the guidelines of Arksey and O’Malley (2005) and Levac et al. (2010), and it was prepared according to the checklist of PRISMA-ScR (Preferred Reporting Items for Systematic Reviews and Meta-Analyses Protocols Extension for Scoping Reviews; Tricco et al. 2018; see Supplementary Appendix A). A systematic scoping review is one of the most suitable methodologies to prepare an integrative and non-interpretative synthesis of a broad research field (Grant and Booth 2009, Schick-Makaroff et al. 2016) and has the further advantage of helping researchers in assessing the extent of the available evidence, organizing it into groups and highlighting gaps in the investigations (Tricco et al. 2018).

In particular, the current systematic scoping review is characterized by five mandatory steps, which are briefly outlined below. A detailed description of the methods is included in Supplementary Appendix B.

The first step regarded the formulations of the research questions:

1. What are the joint action tasks used in EEG-hyperscanning studies?
2. What are the analytical approaches used to study INC?
3. What are the key findings associated with INC?

To answer these research questions, we introduced a new operational definition of joint action based on the theoretical framework developed by Clark (1996) in his studies on verbal joint actions:

Joint action is any form of interpersonal interaction whereby two or more individuals coordinate their participatory actions in space and time to achieve a shared and public goal.

The second step consisted in defining the search strategy. Multiple bibliographic databases of different scientific disciplines were used to select the studies of interest for the present review. Several keywords were initially used. At the end of an iterative process, two independent search terms were retained: ‘hyperbrain’ OR ‘hyperscanning’.

The third step involved the selection of relevant studies, i.e. only hyperscanning studies of joint action performed with EEG and published until August 31st, 2023. This selection was performed by carefully screening the abstracts and full texts according to specific inclusion and exclusion criteria (see Supplementary Appendix B).

**Figure 1.**
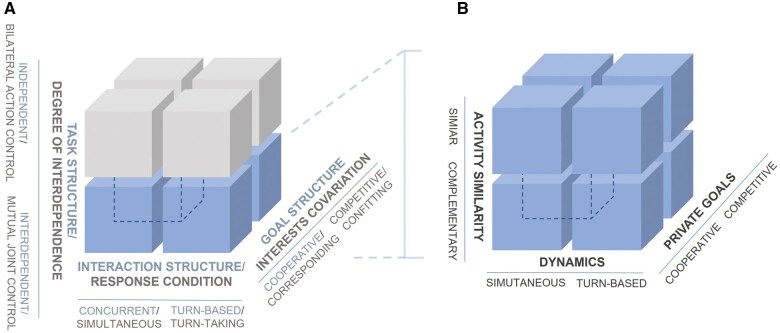
Categorization of the non-verbal joint action: (A) graphical representation of the similarities between the categorization model of interpersonal interactions proposed by Liu and Pelowski (2014b) (text before the slash) and characteristics of the task used by Kelley et al. (2003) in their Atlas of Interpersonal Situation (text after the slash). The four upper cubes in the left image refer to INDEPENDENT interpersonal interactions, while the four lower cubes refer to INTERDEPENDENT interpersonal interactions. (B) Graphic representation of the categorization model of non‑verbal joint actions based on the characteristics of the task proposed in the present systematic scoping review. Dashed lines indicate that joint action is a specific sub‑category of interpersonal interactions, which corresponds to INTERDEPENDENT interaction (represented by the four lower cubes in the left image).

The fourth step regarded the extraction of the relevant information. To this aim, a definition of joint action was mandatory but not sufficient: a classification system that delineates and categorizes the various types of joint action based on fundamental task characteristics was required. Therefore, we propose a new classification system (graphically represented in Fig. 1) in accordance with the theoretical framework of Clark (1996):

1. Cooperative or competitive joint action (based on the private interests of interacting individuals).
2. Simultaneous or turn-based joint action (based on the specific temporal structure of the interaction dynamics).
3. Similar or complementary joint action (based on the similarity of the activities performed by the interacting individuals).

After having categorized the selected studies, we extracted the relevant information according to the defined research questions, including the specific type of joint action task used, the number of interacting individuals, the metrics employed to quantify INC, and the related main findings in EEG patterns and cortical areas involved in joint action.

The fifth and last step involved the presentation of the results in a structured manner, aligning with the research questions outlined earlier. The gathered information is reported according to:

1. The joint action categories investigated, distinguishing between cooperative and competitive, simultaneous and turn-based, similar and complementary joint actions, and between dyadic, triadic, and group-based (> 3 interacting individuals) joint actions.
2. The joint action tasks employed to implement the respective joint action categories.
3. The metrics used to quantify INC, distinguishing between those using phase synchrony, amplitude/envelope correlation, coherence, causality, graph theory and other approaches.
4. The main findings on INC, focusing on the main frequency bands and cortical areas reported to be involved within each category and joint action task.

## Results

The initial search identified 1648 articles. After removing 1064 duplicates, 584 articles focusing on hyperscanning were included for abstract screening. Of these, 409 articles were excluded because they: did not describe experimental studies (e.g. reviews, theoretical articles/book chapters; 167 articles); employed a hyperscanning technique different from EEG (218 articles: 173/36/9 studies used NIRS/MRI/MEG); described studies involving non-human subjects (4 articles); were not retrievable online (10 articles); were not written in English (10 articles).

The retained 175 articles on EEG-hyperscanning underwent full-text screening. Of these, 123 articles were excluded because: the tasks used in the study paradigm did not involve participatory actions and/or a shared public goal; the task involved spoken communication; the study population included children, elderly, and/or non-healthy populations.

**Figure 2.**
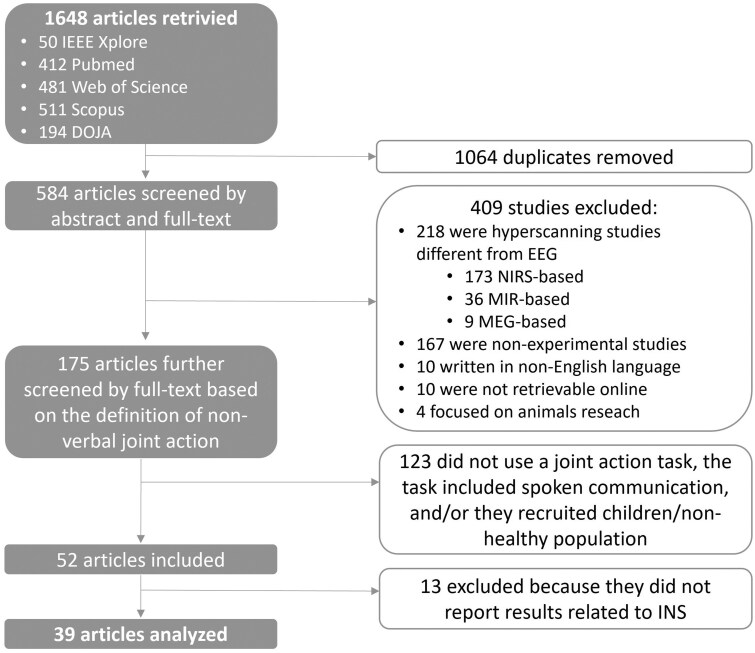
Flow chart of the study selection employed for the given scoping review. Note that our search using additional publications or grey literature, hand searching of reference lists, Google Scholar, and academic social networks (i.e. Research Gate) did not provide relevant results in addition to the listed databases.

Of the selected 52 articles describing EEG-hyperscanning studies with an experimental task consistent with our operational definition of joint action, 13 articles were excluded because the authors did not report results related to INC. As a final result, 39 articles were included in the current systematic scoping review. Figure 2 summarizes the selection procedure, and Table 1 provides a list of the selected articles. Interestingly, the number of EEG-hyperscanning studies consistent with our working definition of joint action has doubled every 5 years since the study of Lindenberger et al. in 2009.

**Table 1.** List of the selected EEG-hyperscanning studies investigating non-verbal joint action, sorted by year of publication.

	Citations	Paradigm	Task	Private goal	Dynamics	Action similarity	Interacting individuals
1	Lindenberger et al. (2009)	Music	Guitar	Coop.	Simult.	Similar	2
2	Astolfi et al. (2011)	Simulation	Piloting	Coop.	Simult.	Compl.	2
3	Astolfi et al. (2012)	Simulation	Piloting	Coop.	Simult.	Compl.	2
4	Naeem et al. (2012)	Movement	Tapping	Coop.	Simult./Turn	Similar	2
5	Sänger et al. (2012)	Music	Guitar	Coop.	Simult.	Compl.	2
6	Dodel et al. (2013)	Computer	Gaming	Coop.	Simult.	Compl.	2
7	Müller et al. (2013)	Music	Guitar	Coop.	Simult.	Similar	2
8	Sänger et al. (2013)	Music	Guitar	Coop.	Simult.	Similar	2
9	Astolfi et al. (2014)	Computer	Gaming	Coop.	Simult.	Similar	2
10	Konvalinka et al. (2014)	Movement	Tapping	Coop.	Simult.	Similar	2
11	Müller and Lindenberger (2014)	Movement	Kissing	Coop.	Simult.	Similar	2
12	Toppi et al. (2015)	Computer	Gaming	Coop.	Simult.	Similar	2
13	Strang et al. (2015)	Computer	Gaming	Coop.	Simult.	Compl.	2
14	Filho et al. (2015)	Movement	Juggling	Coop.	Simult.	Similar	2
15	Sinha et al. (2016)	Computer	Gaming	Coop./Comp.	Turn	Similar	2
16	Toppi et al. (2016)	Simulation	Piloting	Coop.	Simult.	Compl.	2
17	Hemakom et al. (2018)	Computer	Gaming	Coop.	Simult.	Similar	2
18	Kawasaki et al. (2018)	Movement	Tapping	Coop.	Turn.	Similar	2
19	Müller et al. (2018)	Music	Guitar	Coop.	Simult.	Compl.	4
20	Müller and Lindenberger (2019)	Music	Guitar	Coop.	Simult.	Compl.	2
21	Stone et al. (2019)	Movement	Juggling	Coop.	Simult.	Similar	2
22	Astolfi et al. (2020)	Computer	Gaming	Coop.	Simult.	Similar	2
23	Dehais et al. (2021)	Simulation	Piloting	Coop.	Simult.	Compl.	2
24	Léné et al. (2021)	Computer	Gaming	Coop./Comp.	Simult.	Similar	2
25	Liu et al. (2021)	Movement	Gaming	Coop./Comp..	Simult.	Similar	2
26	Maÿe et al. (2021)	Computer	Gaming	Coop.	Simult.	Compl.	2
27	Reinero et al. (2021)	Computer	Problem solving	Coop.	Simult.	Similar	4
28	Sciaraffa et al. (2021)	Computer	Gaming	Coop.	Simult.	Similar	2
29	Shiraishi and Shimada (2021)	Movement	Tapping	Coop.	Simult./Turn.	Similar	2
30	Susnoschi Luca et al. (2021)	Computer	Gaming	Coop./Comp.	Simult.	Similar	2
31	Zamm et al. (2021)	Music	Piano	Coop.	Simult.	Similar	2
32	Gugnowska et al. (2022)	Music	Piano	Coop.	Simult.	Compl.	2
33	Müller and Lindenberger (2022)	Music	Guitar	Coop.	Simult.	Similar	2
34	Ramírez-Moreno et al. (2022)	Music	Different instruments	Coop.	Simult.	Compl.	3
35	Wikström et al. (2022)	Computer	Gaming	Coop.	Simult.	Compl.	2
36	Balconi and Angioletti (2023a)	Movement	Tapping	Coop.	Simult.	Similar	2
37	Balconi and Angioletti (2023b)	Movement	Tapping	Coop.	Simult.	Similar	2
38	Müller and Lindenberger (2023)	Music	Guitar	Coop.	Simult.	Similar	4
39	Ogawa and Shimada (2023)	Movement	Object manipulation	Coop.	Simult.	Similar	2

### Joint action categories

As detailed in the Methods section, non-verbal joint action tasks were categorized in cooperative or competitive, simultaneous or turn-based, similar or complementary (cp. Fig. 1). We found that 35 studies investigated cooperative joint actions, whereas only four studies compared cooperation and competition. Thirty-four studies explored simultaneous joint actions, four studies investigated turn-based joint actions, and one study compared simultaneous and turn-based joint actions. Finally, 26 studies explored similar joint actions, whereas 13 studies investigated complementary joint actions. No study compared similar and complementary joint actions (**Table 1**).

**Figure 3.**
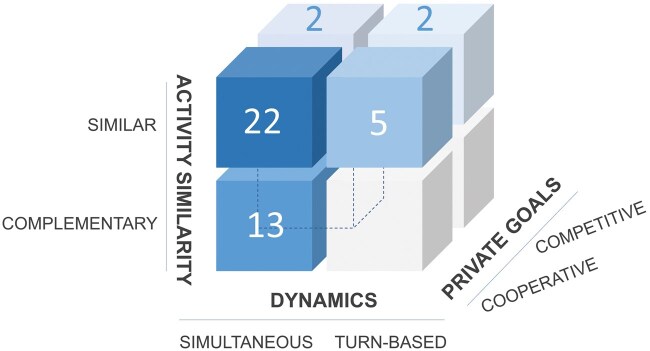
Graphical representation of the explored and unexplored categories of non‑verbal joint action in EEG‑hyperscanning literature. The number of publications identified for each category is given in each respective box.

Considering all possible combinations of these classification properties, EEG-hyperscanning studies have investigated five categories of joint action tasks, whereas three categories remain unexplored. A graphical representation of the categories and their interrelation is shown in Fig. 3. Specifically, the following categories were investigated:

1. Cooperative/simultaneous/similar joint action: 22 times.
2. Cooperative/simultaneous/complementary joint action: 13 times.
3. Cooperative/turn-based/similar joint action: 5 times.
4. Competitive/simultaneous/similar joint action: 2 times.
5. Competitive/turn-based/similar joint action: 2 times.

The three unexplored categories encompass:

1. Cooperative/turn-based/complementary joint action.
2. Competitive/simultaneous/complementary joint action.
3. Competitive/turn-based/complementary joint action.

It is worth noting that the total number of studies across categories adds up to 44, exceeding the number of articles in this review (n = 39). This discrepancy arises because five out of the 39 studies compared two categories of non-verbal joint actions: either cooperative vs. competitive joint action (n = 4) or simultaneous vs. turn-based joint action (n = 1).

Another way to categorize joint action tasks is based on the number of interacting individuals involved. Among the selected studies, 35 studies featured dyadic joint action tasks, one study employed a triadic task, and three studies implemented group-based joint action tasks, each involving four interacting individuals. Dyadic joint action tasks were used across various categories of joint action. Conversely, the triadic joint action task was employed in the context of cooperative/simultaneous/similar joint action (n = 1), whereas the group-based tasks were employed for cooperative/simultaneous/similar (n = 2) and cooperative/simultaneous/complementary (n = 1) joint actions (see Table 1).

### Joint action tasks

The experimental tasks described in the selected articles can be categorized into five main types: those predominantly based on motor behaviour (i.e. movement-based; n = 12), those centred around musical performance (i.e. music-based; n = 11), those utilizing computer-mediated gaming (i.e. computer-based; n = 12), and those simulating real-life situations (i.e. simulation-based; n = 4).

**Figure 4.**
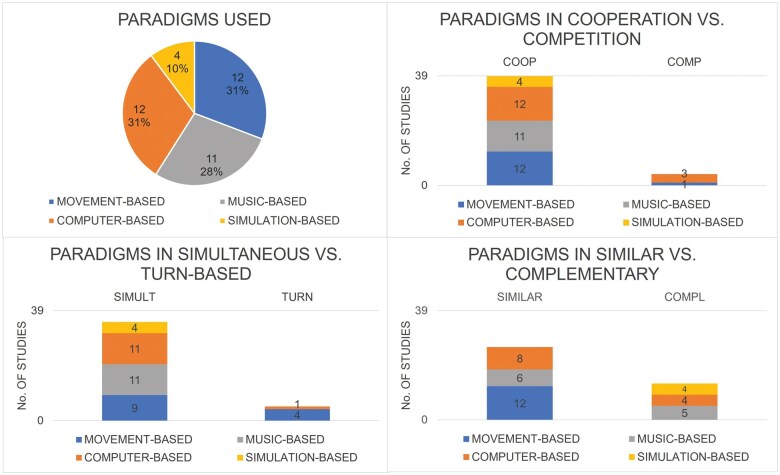
Relative and absolute distributions of experimental paradigm categories in non-verbal joint action studies. In pie-chart (A), the overall distribution of PARADIGMS USED in all studies is displayed, without distinguishing between categories of joint action. The remaining stacked bar chart break down the joint action categories: (B) COOPERATION vs. COMPETITION, (C) SIMULTANEOUS vs. TURN-BASED, (D) SIMILAR vs. COMPLLEMENTARY. The data provide an overview of paradigm prevalence in non-verbal joint action research, highlighting trends, and differences across various study categories.

Among the 12 studies employing movement-based non-verbal joint action tasks, six studies employed finger-tapping synchronization tasks, whereas six studies focused on more complex interpersonal motor tasks involving whole-body movements, including dyadic juggling (n = 2), tennis playing (n = 1), romantic kissing (n = 1), and object manipulation either in a virtual reality environment (n = 1) or in a real-world setting (n = 1). Out of the 11 music-based joint action tasks, eight tasks involved the use of guitars, two tasks employed pianos, and one task required interacting individuals to play in unison by using different music instruments. Of the twelve studies using computer-based gaming tasks, one study employed a brain-computer interface. Finally, the four studies based on simulation joint action tasks employed simulated piloting scenarios. These results are presented in the Table 1 and Fig. 4.

When examining the experimental tasks used within each joint action category, we observed that movement-based tasks were predominantly employed to investigate cooperative joint actions (n = 12), with only one study investigating also competitive joint actions. In both cases, participants engaged in similar actions. Moreover, most studies adopting movement-based tasks favoured simultaneous (n = 9) over turn-based coordination dynamics (n = 4). Within the category of music-based joint actions, the experimental tasks were used to examine cooperative and simultaneous actions (n = 11). Among these studies, individuals either executed similar (n = 6) or complementary actions (n = 5). Computer-based paradigms were used in the cooperative joint action category (n = 12). Interestingly, a remarkable number of studies investigating competitive joint actions (3 out of 4) adopted computer gaming tasks. Lastly, simulation-based tasks were less commonly utilized across all joint action categories (n = 4). However, when applied, these tasks exclusively focused on cooperative/simultaneous/complementary joint actions.

These results are detailed in Fig. 4B for differences between cooperation and competition, in Fig. 4C for differences between simultaneous vs. turn-based tasks, in Fig. 4D for differences between similar vs. complementary joint action categories.

### Analytical approaches to INC

Recently, Hakim et al. (2023) reviewed and described the methods currently used in EEG-hyperscanning studies to quantify INC. The analytical approaches employed to quantify INC can be grouped into six categories, which are reported below with an indication of the number of studies (n =…) in which they were used:

1. Phase synchrony metrics, which quantify the temporal alignment (or delay) of EEG activity in the brains of the individuals involved in joint action (n = 12).
2. Amplitude/envelope correlation metrics, which quantify the linear relationship (i.e. correlation) between the amplitude (or its envelope) of the EEG signals (n = 5).
3. Coherence metrics, which quantify the degree of similarity of the EEG activity in the brains of the individuals involved in joint action at specific frequency bands over given periods of time (n = 7).
4. Causality/directionality metrics, which quantify the causal relationship and/or directionality of information flow within and between the brains of the individuals involved in joint action (n = 9).
5. Graph theoretical metrics, which typify the functional organization of the neural networks active in the brains of the individuals involved in joint action (n = 14). It is worth highlighting that graph theoretical metrics were calculated after the implementation of other methodologies: graph theory metrics were calculated for functional connectivity maps obtained with phase synchrony analyses (nine studies), or after causality/directionality analyses (four studies), or after coherence analysis (1 study).
6. Other approaches, including Mutual Information, Geometrical Models, Logistic Regression, Time-frequency analysis, Intrinsic Synchrosqueezing Coherence (ISC), Multivariate Granger Causality, Covariance, and Bispectrum (n = 8).

It is worth mentioning that 26 out of the selected 39 studies employed one of the six INC metrics types listed above, 11 studies used two of these metrics, one study employed three INC metrics types, and another study employed four types of INC metrics (see Table 2).

**Table 2.** Summary of the INC metrics used in selected EEG-hyperscanning studies investigating non-verbal joint action.

N	Citations	Analytical approaches to quantify INC							
		Phase synch.	Amplit./envelope	Cohe-rence	Caus./direct.	Graph theory			Other
						Phase synch.	Cohe-rence	Caus./direct.	
1	Lindenberger et al. 2009	PLI; IPC							
2	Astolfi et al. 2011				PDC				
3	Astolfi et al. 2012				PDC				
4	Naeem et al. 2012								MI
5	Sänger et al. 2012	IPC				S; CPL; CC; M			
6	Dodel et al. 2013								GM
7	Müller et al. 2013	PSI; ACI			ICI	S; Deg; CPL; CC; SWC; M (wmDeg; PC)			
8	Sänger et al. 2013							S	
9	Astolfi et al. 2014				PDC	D; DV			
10	Konvalinka et al. 2014								LG
11	Müller and Lindenberger 2014					S; CPL; CC; SWC; M (wmDeg; PC); GE; LE			
12	Toppi et al. 2015							D; CPL; CC; GE; LE	
13	Strang et al. 2015								TF
14	Filho et al. 2015			Coh			CPL; CC; SWC		
15	Sinha et al. 2016		PCC						
16	Toppi et al. 2016				PDC	ICD; DD; IALD			
17	Hemakom et al. 2018								ISC
18	Kawasaki et al. 2018	PSI	CrosC						
19	Müller et al. 2018				aICI			CPL; CC; SWC; GE; LE	
20	Müller and Lindenberger 2019				aICI			S; M	
21	Stone et al. 2019					CPL; CC; SWC; GE; D; LAT; RA; IIR			
22	Astolfi et al. 2020				PDC	Deg; S; CPL; CC; D; DV; M; GE; LE			
23	Sciaraffa et al. 2021								mvarGC
24	Reinero et al. 2021			Coh					
25	Dehais et al. 2021					GE			COV
26	Léné et al. 2021	PLV		WTC					
27	Liu et al. 2021	PLV	PCC						
28	Maÿe et al. 2021	PLV	AC; PC	CircC	PDC; DTF				
29	Shiraishi and Shimada 2021	PSI							
30	Susnoschi Luca et al. 2021	PLV							
31	Zamm et al. 2021		AEC						
32	Gugnowska et al. 2022	PLV							
33	Müller and Lindenberger 2022	PLI							
34	Ramírez-Moreno et al. 2022								Bis
35	Wikström et al. 2022	CCorr							
36	Balconi and Angioletti 2023a			PCC					
37	Balconi and Angioletti 2023b			PCC					
38	Müller and Lindenberger 2023					S; CPL; CC, GE; LE			
39	Ogawa and Shimada 2023			TI					

Total		**12**	**5**	**7**	**9**	**9**	**1**	**4**	**8**
						14			

Phase synchrony metrics—PLI = phase lag index; IPC = interbrain phase coherence; PSI = phase synchronization index; ACI = absolute coupling index; PLV = phase locking value; CCorr = circular correlation coefficient. Amplitude/envelope correlation measures—PCC = Pearson correlation coefficient; CrosC = cross-correlation; AC = amplitude correlation; PC = power correlation; AEC = amplitude/envelope correlation. Coherence measures—Coh = coherence; WTC = wavelet transform coherence; CircC = circular coherence; PCC = partial correlation coefficient; TI = total interdependence. Causality/directionality measures—PDC = partial directed coherence; ICI = integrative coupling index; aICI = adaptive ICI; DTF = directed transfer function. Graph-theory measures—S = strength; CPL = characteristic path length; CC = clustering coefficient; M = modularity; Deg = degree; SWC = small-world coefficient; wmDeg = within module degree; PC = participation coefficient; D = density; Div = divisibility; GE = global efficiency; LE = local efficiency; ICD = inter-connections density; DD = directed density; IALD = inter-areas link density; LAT = lateralization; RA = regional asymmetry; IIR = intra-brain/inter-brain ratio. Other measures—MI = mutual information; GM = geometrical model; LG = logistic regression; TF = time-frequency analysis; ISC = intrinsic synchrosqueezing coherence; mvarGC = multivariate Granger causality; COV = covariance; bis = bisprectum

It is important to note that, prior to calculating INC metrics, almost all studies applied the following data transformation or decomposition methods: Gabor transform (n = 8), Morlet transform (n = 3), Hilbert transform (n = 6), Hanning-taper transform (n = 3), Fast Fourier transform (FFT; n = 6), multivariate models (MVAR; six studies), and noise-assisted multivariate empirical mode decomposition (NA-MEMD; one study). Six studies solely applied filtering as a pre-processing step before quantifying INC.

### Key findings

In the following, key findings on INC are presented with reference to the types of data analysis approach employed. For each data analysis approach, the findings are grouped with reference to the joint action categories and experimental task types according to our novel categorization. This approach allowed us to provide a comprehensive view of the key findings, facilitating the comparison between different joint action categories and tasks in relation to a specific data analysis approach.

#### Phase synchrony measures

Phase synchrony measures were employed 12 times to investigate INC across all joint action categories (Lindenberger et al. 2009, Sänger et al. 2012, Müller et al. 2013, Kawasaki et al. 2018, Léné et al. 2021, Liu et al. 2021, Maÿe et al. 2021, Shiraishi and Shimada 2021, Susnoschi Luca et al. 2021, Gugnowska et al. 2022, Müller and Lindenberger 2022, Wikström et al. 2022).

##### Cooperative/simultaneous/similar joint action category

A phase synchrony metric was employed in six studies belonging to this category. One study employed a movement-based task (Shiraishi and Shimada 2021), three studies employed music-based tasks (Lindenberger et al. 2009, Müller et al. 2013, Müller and Lindenberger 2022), and two studies employed computer-based games (Léné et al. 2021, Susnoschi Luca et al. 2021). Shiraishi and Shimada (2021) employed a sequential tapping task and found high INC in the delta band between the central and occipital regions of the individual who initiated the task (i.e. leader) and the parietal and occipital regions of the individual who followed (i.e. follower). However, there were no significant differences with the control group (i.e. pseudo-pair dyad combinations). Lindenberger et al. (2009) examined whether the INC between two guitar players was enhanced during the preparatory period for setting the metronome tempo and while playing a melody together. During the preparatory period, strong INC was found in the fronto-central areas within the frequency range of 3–8 Hz, with a peak at 5 Hz. During the musical performance, a robust INC was found in the same areas active during the preparatory period, but in the frequency range of 0.5–7.5 Hz, with a peak at 3.3 Hz. This coupling disappeared after the end of the performance. In a subsequent study on joint guitar playing, Müller et al. (2013) found strong INC in the frontal and central regions of the two musicians at 6 Hz. Similarly, Müller and Lindenberger (2022), who investigated INC between the brains of two guitarists during metronome tempo setting and musical performance, found strong INC in the delta and theta frequency bands in the fronto-central areas, particularly evident after the onset of the musical performance. Léné et al. (2021) measured INC between two individuals engaged in a computer-based fast button response task performed under cooperative and competitive conditions. The results showed a higher INC in the theta frequency band in the right parietal and occipital areas during cooperation as compared to competition. However, authors highlighted that phase synchronization approaches were less suitable than other approaches (i.e. coherence) in capturing INC in this type of task. Lastly, Susnoschi Luca et al. (2021) measured INC while two individuals interacted during a multiuser alpha neurofeedback-based gaming session. Overall, cooperative gaming led to robust INC in the fronto-central theta band, especially on the right hemisphere, in the centro-posterior alpha band, and in the parieto-occipital and fronto-central beta band.

##### Cooperative/simultaneous/complementary joint action category

Four studies employed phase synchrony metrics to quantify INC among musicians playing different instruments (Sänger et al. 2012, Gugnowska et al. 2022) or individuals playing a computer-based game (Maÿe et al. 2021, Wikström et al. 2022). Sänger et al. (2012) observed high INC between pianists during the preparatory period, and before and after musical performance onsets. This INC was more prominent in frontal and central than parietal areas and was more pronounced in the delta than theta frequency band. Gugnowska et al. (2022) investigated INC during a joint piano performance. Their findings revealed stronger INC in the gamma frequency band when pianists were unfamiliar with the musical parts of their partners as compared to when they were familiar with them. Additionally, stronger INC was observed at the beginning of the first musical phase in the delta and theta bands when pianists had received incongruent tempo instructions. Maÿe et al. (2021) investigated INC between two individuals engaged in a computer gaming task without finding any statistically significant effects. On the other hand, Wikström et al. (2022) found significant INC in all frequency bands and cortical areas examined (i.e. alpha, beta, gamma bands in frontal, central, parietal, occipital areas) while two individuals, positioned in different rooms, were engaged in a computer-based video game. Notably, this synchrony decreased during the playing sessions, although it was found to be higher during the second experimental (i.e. playing) session compared to the first one.

##### Cooperative/turn-based/similar joint action category

Three studies, all based on movement-based tasks, utilized phase synchrony metrics to investigate INC (Kawasaki et al. 2018, Liu et al. 2021, Shiraishi and Shimada 2021). Kawasaki et al. (2018) investigated INC between pairs of individuals engaged in an alternating tapping task, comparing pairs that performed well with those exhibiting poor performance. The authors found alpha INC in the posterior brain areas only in the group of individuals who were good at coordinating their tapping rhythms with their partner. This INC was observed not only during task performance but also immediately before and after tapping onset. Similarly, Shiraishi and Shimada (2021) employed an alternating tapping task and found high INC in the theta band between the right frontal region of the individual who initiated the task (‘the leader’) and the right temporal-parietal region of the individual who followed (‘the follower’). Finally, Liu et al. (2021) explored INC between two individuals playing a motion-sensing sports video game (i.e. tennis). In this study, the authors did not find any significant results.

##### Competitive/simultaneous/similar joint action category

Two studies employed phase synchrony metrics to investigate INC (Léné et al. 2021, Susnoschi Luca et al. 2021). Both studies were mentioned earlier. Léné et al. (2021) compared cooperation and competition but did not found any difference in INC. Susnoschi Luca et al. (2021) reported a higher inter-hemispheric asymmetry in the theta band during competition as compared to cooperation, with a greater involvement of the left cortical area as compared to the right one. Furthermore, alpha INC during competition primarily occurred between the parietal and occipital areas and the frontal area, with less engagement of midline areas compared to cooperation. Finally, beta INC during competition predominantly involved the fronto-central and parieto-occipital areas, like during cooperation.

##### Competitive/turn-based/similar joint action category

Here, there is only the study by Liu et al. (2021) described earlier and employing a movement-based task. The authors reported no significant results in INC.

#### Amplitude/envelope correlation measures

Amplitude/envelope correlation measures were employed 5 times to investigate INC across all joint action categories (Sinha et al. 2016, Kawasaki et al. 2018, Liu et al. 2021, Maÿe et al. 2021, Zamm et al. 2021).

##### Cooperative/simultaneous/similar joint action category

Only Zamm et al. (2021) employed amplitude/envelope correlation to investigate INC during a piano duet performance. Their results revealed that the two pianists exhibited INC throughout the entire music performance. Furthermore, this INC was particularly evident when the music tones were produced during the performance as opposed to silent transition phases.

##### Cooperative/simultaneous/complementarity joint action category

Amplitude/envelope correlation was employed to investigate INC in only one study on two individuals playing with a computer gaming task (Maÿe et al. 2021). However, the authors reported no significant result.

##### Cooperative/turn-based/similar joint action category

For this category, three out of five studies utilized amplitude/envelope correlation to measure INC (Sinha et al. 2016, Kawasaki et al. 2018, Liu et al. 2021). Kawasaki et al. (2018) examined INC between pairs of individuals engaged in an alternating tapping task. They compared pairs with good coordination performance to those with poor performance. INC was higher in the alpha band in the fronto-central areas, but only for individuals who were skilled at coordinating their tapping rhythms. The other two studies compared INC of cooperative and competitive joint actions in the context of turn-based/similar tasks (Sinha et al. 2016, Liu et al. 2021). In Sinha et al. (2016), a computerized pong-game was employed in both cooperative and competitive conditions implemented in both physical and virtual spaces. The cooperation scenario showed significantly higher INC in the alpha band as compared to the competing scenario. Furthermore, cooperation in a virtual space enhanced INC across all frequency bands as compared to cooperation in a physical space. Lastly, Liu et al. (2021) used a motion-sensing tennis game and found that, during cooperative gameplay, INC was observed in a right-lateralized fronto-central area in the delta band. Additionally, INC was observed in a widespread area, particularly in the theta band. In contrast, during competitive gameplay, INC was observed primarily in the occipital areas in the alpha and beta bands. Importantly, the authors highlighted that amplitude/envelope correlation is more suitable than phase synchrony in capturing INC dynamics in this type of task.

#### Coherence measures

Coherence measures were employed 7 times across three joint action categories: Cooperative/simultaneous/similar (Filho et al. 2016, Léné et al. 2021, Maÿe et al. 2021, Reinero et al. 2021, Ogawa and Shimada 2023, Balconi and Angioletti 2023a, 2023b), cooperative/simultaneous/complementary (Maÿe et al. 2021), and competitive/simultaneous/similar joint actions (Léné et al. 2021).

##### Cooperative/simultaneous/similar joint action category

Coherence measures were used in four experimental tasks involving movement (Filho et al. 2016, Ogawa and Shimada 2023, Balconi and Angioletti 2023a; 2023b), and in two computer games (Léné et al. 2021, Reinero et al. 2021). Filho et al. (2016) investigated INC between two individuals engaged in a dyadic juggling task at different difficulty levels. The results indicated INC in the alpha and theta bands across various brain areas, including frontal, central, parietal, and occipital regions. Notably, this INC tended to diminish as task difficulty increased. Balconi and Angioletti (2023a, 2023b) investigated INC between two individuals engaged in finger tapping. The study was performed under two distinct interoceptive conditions, wherein interacting individuals either focused on their breath or did not focus on their breath. Both studies reported greater INC during task performance in the delta, theta, and alpha bands, particularly in the frontal and central brain areas, more specifically in the condition where participants were instructed to focus on their breath. Ogawa and Shimada (2023) explored INC between two individuals engaged in a cooperative motor task, which involved moving blocks in a shared virtual reality environment. The authors reported higher levels of INC across frequency bands (i.e. theta, alpha, beta) and cortical areas (especially frontal and central areas) during cooperation as compared to when the same task was performed individually. In another study, Reinero et al. (2021) investigated INC among four individuals engaged in collaborative problem-solving using a computer. In this study, the authors did not find significant results in the experimental group (i.e. interactive task) as compared to the control condition (i.e. individual task). However, they reported a reduction in INC over time. On the other hand, Léné et al. (2021) measured INC among individuals engaged in a computer-based cooperative fast button response task performed under cooperative, competitive, and individual conditions, and found higher INC in the frontal alpha and parietal theta bands during cooperation.

##### Cooperative/simultaneous/complementary joint action category

Only Maÿe et al. (2021) employed coherence to investigate INC between two individuals engaged in a computer gaming task. However, they did not find any significant results.

##### Competitive/simultaneous/similar joint action category

Only Léné et al. (2021) employed coherence to investigate INC among individuals engaged in a computer-based fast button response task performed under cooperative, competitive, and individual conditions (see above). During competition, they found increased theta INC in parietal and occipital areas and increased alpha INC in the parietal area.

#### Causality/directionality measures

Nine studies employed causality/directionality measures to quantify INC in two joint action categories, namely cooperative/simultaneous/similar (Müller et al. 2013, Astolfi et al. 2014, 2020), and cooperative/simultaneous/complementary (Astolfi et al. 2011, 2012, Toppi et al. 2016, Müller et al. 2018, Müller and Lindenberger 2019, Maÿe et al. 2021).

##### Cooperative/simultaneous/similar joint action category

Three studies employed causality/directionality measures to investigate INC and directional patterns among musicians engaged in a group performance (Müller et al. 2013) and among individuals playing a computer game (Astolfi et al. 2014, 2020). Müller et al. (2013) found specific directional patterns in INC among improvising guitarists. These directional patterns involved frontal and parietal alpha oscillations but varied depending on the individual’s role as leader or follower. Astolfi et al. (2014) examined INC patterns among individuals playing computer games and found a significant increase of INC in the theta frequency band when interacting with a human compared to playing with the computer or alone. In their subsequent study, Astolfi et al. (2020) reported similar results, but this time referring to all frequency bands. However, in both studies, the authors did not observe specific directional patterns.

##### Cooperative/simultaneous/complementary joint action category

Causality/directionality measures were employed to investigate INC and directional patterns among musicians engaged in a group performance (Müller et al. 2018, Müller and Lindenberger 2019), in individuals playing a computer game (Maÿe et al. 2021), and in individuals simulating a piloting task (Astolfi et al. 2011, 2012, Toppi et al. 2016). Müller et al. (2018) observed widespread INC across all cortical areas of the musicians involved in group performance, particularly in the delta and theta bands. They identified different directional patterns that varied depending on the guitarist playing from sheet music. In a subsequent study, Müller and Lindenberger (2019) reported similar results in two guitarists who were improvising. In their study on two individuals playing a computer game, Maÿe et al. (2021), in addition to the other approaches described earlier, employed a causality/directionality approach to investigate INC. Like the results obtained with the other approaches, they reported no significant results. Astolfi et al. (2011, 2012) studied INC in piloting simulation tasks, which involve various roles and different flight phases with varying levels of interaction (i.e. independent and interdependent). They found higher INC during interaction phases as compared to non-interactive phases. This INC was predominant in the frontal and parietal alpha band, with different directional patterns depending on the role and flight phase. Finally, Toppi et al. (2016) extended these findings by also identifying the involvement of frontal and parietal theta oscillations.

#### Graph theory measures

Fourteen studies employed graph theoretical measures to study INC from a network perspective within the cooperative/simultaneous/similar (Müller et al. 2013, Sänger et al. 2013, Astolfi et al. 2014, 2020, Müller and Lindenberger 2014, 2023, Toppi et al. 2015, Filho et al. 2016, Stone et al. 2019) and cooperative/simultaneous/complementary joint action categories (Sänger et al. 2012, Toppi et al. 2016, Müller et al. 2018, Müller and Lindenberger 2019, Dehais et al. 2021).

##### Cooperative/simultaneous/similar joint action category

Graph theoretical measures were employed to characterize INC among individuals engaged in movement-based tasks (Müller and Lindenberger 2014, Filho et al. 2016, Stone et al. 2019), musical performance (Müller et al. 2013, Sänger et al. 2013, Müller and Lindenberger 2023), and computer gaming (Astolfi et al. 2014, 2020, Toppi et al. 2015). Graph theoretical measures were applied following a functional connectivity analysis based on phase synchrony, except in one study that relied on coherence analysis (Filho et al. 2016). Müller and Lindenberger (2014) investigated INC during romantic kissing. Their study revealed INC in the theta and alpha frequency bands, with network strengths being higher and characteristic path lengths (CPL) shorter when individuals were engaged in romantic kissing compared to when they were kissing their own hand. In another study, Filho et al. (2016) investigated the topological features of INC during an interactive juggling task with different difficulty levels. Their results showed higher values of CPL and lower values of clustering coefficient (CC) across frontal, central, parietal, and occipital cortical areas in the theta and alpha frequency bands, at all difficulty level. Furthermore, the small-word coefficient (SWC) was always lower than unit in both frequency bands, and its values decreased when difficulty increased. In contrast, Stone et al. (2019), who compared less and more skilled jugglers, did not find any significant results, although reporting a trend suggesting that less skilled jugglers exhibited right-lateralized INC, whereas more skilled jugglers displayed greater left lateralized INC. In the context of music performance, Müller et al. (2013) observed that the degree and strength of the functional network connections increased at lower frequencies and were most pronounced at frontal sites while musicians were playing. The study revealed increased values of CC and decreased values of CPL in the beta band that implied an increase in SWC. Moreover, network modularity revealed a significant increase at higher frequencies. In another study, Sänger et al. (2013) found that INC networks strength in musicians varied as a function of the musical roles of leader and follower, being higher in certain brain areas (e.g. frontal and parietal, respectively for leader and follower) and frequency bands (i.e. alpha and beta for both roles). Lastly, Müller and Lindenberger (2023) found that INC network strength was higher in the quartette playing music than in the audience listening the performance. These changes in INC strength were accompanied by changes in the INC network topology, with higher values of CC, global (GE) and local efficiency (LE) and decreased CPL in the quartet compared to the audience. The values of all these measures changed as a function of frequency bands. Astolfi et al. (2014) used measures of interpersonal neural density and divisibility to investigate how two brains organize during a computer gaming task performed cooperatively with either a human or a computerized player or performed individually. Results showed higher network density and reduced divisibility in the joint condition compared to the control and individual conditions. Toppi et al. (2015) used the same task and found higher GE during the joint action compared to the individual condition. In another study performed using the same task, Astolfi et al. (2020) found higher values of network density, GE, LE and CC, and lower values of divisibility and modularity when the level of interaction between individuals increased. This was found in all frequency bands, although lower frequencies (theta and alpha) showed higher levels of GE, LE and lower levels of modularity with respect to higher frequency bands (beta and gamma).

##### Cooperative/simultaneous/complementary joint action category

The graph theory-based approach was used to investigate INC among individuals engaged in musical performance (Sänger et al. 2012, Müller et al. 2018, Müller and Lindenberger 2019) and piloting simulation (Toppi et al. 2016, Dehais et al. 2021). Within this joint action category, only one study used graph theoretical measures on functional networks based on phase synchrony (Sanger et al. 2012), whereas the graph theoretical approach relied on analyses of causality/directionality in all other cases. Sänger et al. (2012) revealed enhanced network strengths and CPL and lower CC during preparatory tempo setting and after the play onset. Moreover, the INC network topology varied as a function of the musical roles of leader and follower. Müller et al. (2018) studied INC networks in a guitar quartet. They reported increased strength in lower frequencies and higher CPL, CC and LE and lower GE at higher frequencies. Additionally, they found that the INC networks underwent structural changes over time, often influenced by the musical context. Similar results were found in Müller and Lindenberger (2019), who examined INC networks during guitar duo improvisation. They observed that coupling strengths and community structures exhibited varying patterns depending on the frequency band and musical situation. In piloting simulation, Toppi et al. (2016) investigated INC networks in two individuals engaged in interactive and non-interactive phases, revealing dense patterns (higher density) of INC during the interactive flight phases, primarily linking frontal and parietal brain areas in the theta and alpha bands, whereas INC approached zero during the non-interactive phases. Dehais et al. (2021) also investigated INC networks among individuals engaged in aircraft piloting simulation and observed high GE when individuals cooperated as compared to when they did not cooperate.

#### Other approaches

Eight studies investigated INC using a data analysis approach not included in the previous categories (Naeem et al. 2012, Dodel et al. 2013, Konvalinka et al. 2014, Strang et al. 2015, Hemakom et al. 2018, Dehais et al. 2021, Sciaraffa et al. 2021, Ramírez-Moreno et al. 2022).

##### Cooperative/simultaneous/similar joint action category

Three studies employed various approaches within this joint action category. Konvalinka et al. (2014) used a synchronized finger-tapping task and, through the application of a power-based multivariate classification system performed using logistic regression, demonstrated a strong suppression of alpha and low-beta oscillations over the motor and frontal areas. They also observed asymmetric patterns of frontal alpha-suppression in each pair of interacting individuals, occurring during both task anticipation and execution, where only one member exhibited a frontal component. Sciaraffa et al. (2021) employed a Granger causality-based multivariate classification to investigate INC in individuals engaged in the construction of a 3D model. They identified a specific involvement of frontal beta oscillations during joint action. Hemakom et al. (2018) investigated INC among two individuals engaged in a computer game (i.e. bar balancing) at different difficulty levels by using a specific time-frequency measure known as intrinsic synchrosqueezing coherence. They observed that INC was robust in the theta band and that synchrony tended to change (lower theta decrease and upper theta increase) as task difficulty increased.

##### Cooperative/simultaneous and turn-based/similar joint action categories

These two categories are grouped because there is only one study by Naeem et al. (2012) employing an entropy-based analysis (Mutual Information) to investigate the INC in individuals performing a finger-tapping task in both simultaneous and turn-based modality. The authors did not find any significant results in the case of simultaneous task whereas, in the turn-based condition, they observed a pronounced involvement of frontal, occipital, and parietal alpha oscillations in the INC topography.

##### Cooperative/simultaneous/complementary joint action category

Four studies employed various approaches within this joint action category. Ramírez-Moreno et al. (2022) studied INC among three musicians engaged in a jazz improvisation using a Bispectrum approach. They observed that INC increased when more musicians were performing as compared to when only one musician was playing and the others were listening. This increase was most prominent in the beta and gamma bands and occurred in the temporal, occipital, and parietal areas. They also found higher INC during synchronized musical performance and lower INC during desynchronized performance. Dodel et al. (2013) employed an approach based on geometric models to explain brain dynamics variability in geometric terms and categorized its sources into detrimental and non-detrimental to performing a computer-based gaming task. Through this approach, they identified a low-dimensional subspace of INC dynamics in the beta and gamma frequency bands across all cortical areas of the interacting individuals. Strang et al. (2015) investigated INC among individuals playing a computer game under different conditions (i.e. low and high task load) employing time-frequency measures. In this study, the authors found frontal theta INC, which was primarily driven by the team-task environment. Furthermore, they found that during the low task load condition, INC was higher compared to when the task load was more demanding. Finally, Dehais et al. (2021) investigated INC in individuals engaged in aircraft piloting simulation by using covariance. In this study, the authors found greater INC in the theta and alpha frequency bands.

## Discussion

### Joint action categories

The growing interest in EEG-hyperscanning investigations of non-verbal joint action is witnessed by the increasing number of published research during the last years. The main focus was clearly on understanding INC underlying cooperation, whereas competition received relatively less attention, likely because interpersonal coordination is more naturally realized during cooperative tasks. In fact, cooperation is a common aspect of human interaction and its study permits gaining insights into the neural basis of social bonding (Kinreich et al. 2017, Berendzen 2023), attachment (Djalovski et al. 2021), empathy (Goldstein et al. 2018, Peng et al. 2021, Troncoso et al. 2023), and teamwork (Filho et al. 2015, Dikker et al. 2021), which are relevant in various contexts, from everyday life to professional settings (Balconi et al. 2020). Therefore, the design of cooperative joint action experiments was preferred and allowed capturing the neural dynamics of cooperation in a controlled setting.

In terms of coordination dynamics, simultaneous joint actions have received much more attention than turn-based joint actions. In fact, simultaneous joint action tasks involve individuals acting in coordination with each other simultaneously, akin to the fluid nature of many everyday social activities. Researchers may prioritize these tasks because they offer greater experimental control as they allow to precisely time and synchronize the interacting individuals’ actions, facilitating the analysis of the neural data related to the coordination process. In contrast, turn-based joint actions are characterized by pauses or breaks between individual actions, which are—in general—alternated (Liu et al. 2017). These intermittent periods of coordination introduce additional complexities in data analysis and interpretation (Zamm et al. 2023b). Researchers need to account for the temporal gaps between actions, which can vary in duration and can potentially impact the coordination dynamics. Therefore, although turn-based joint actions can also be precisely timed and synchronized, their intermittent nature introduces an additional layer of complexity to be considered when analysing the neural data.

In terms of activity similarity, more attention was dedicated to paradigms where individuals engage in similar actions as opposed to those involving complementary actions. The greater emphasis on similar actions is likely due to the possibility of directly comparing the neural activity of individuals engaged in similar actions.

Lastly, most studies focused on dyadic joint action, which have been shown to be remarkably versatile, spanning across different categories of non-verbal joint action. Much fewer studies investigated joint action among multiple individuals. The prevalence of dyadic EEG-hyperscanning studies on joint action probably stems from the need to simplify interactions, ensure rigorous experimental control, overcome technical limitations, and reduce data analysis complexity. However, it is worth noting that challenges related to studying interactions in larger groups are gradually being addressed with ongoing advancements in technology and research methodologies, as shown by different authors (Müller et al. 2018, Reinero et al. 2021, Ramírez-Moreno et al. 2022, Müller and Lindenberger 2023, Tamburro et al. 2023).

### Joint action tasks

Joint actions have been investigated by implementing a variety of tasks, each characterized by advantages and disadvantages, as discussed in the following paragraphs.

#### Movement-based tasks

Movement-based tasks have been most used to study joint action, encompassing simple movements, such as finger-tapping synchronization (e.g. Shiraishi and Shimada 2021, Balconi and Angioletti 2023a, 2023b) and more complex whole-body movements, as in dyadic juggling (Filho et al. 2016, Stone et al. 2019). While simple movement-based joint action paradigms offered precise control over the experiments, complex movement-based paradigms were more suitable to mimic real-world situations. From a real-world perspective of joint action neuroscience, complex movement-based joint action paradigms should be preferred (Kasai et al. 2015, Krugliak and Clarke 2022). However, balancing experimental control and ecological validity is an ongoing challenge in joint action research (Ladouce et al. 2016, Matusz et al. 2019). In fact, ecological paradigms involving free full-body movements pose a crucial analytical question regarding the removal of motion-related artefacts, which are difficult to remove from EEG signals without altering brain activity signals because they do not show a stereotyped structure. Indeed, only few attempts have been made so far to develop reliable methods for removing motion-related artefacts (Mihajlović et al. 2014, Gorjan et al. 2022). An interesting solution in this context has been proposed by researchers who used technology to augment (Liu et al. 2021) or virtually reproduce reality (Ogawa and Shimada 2023).

Importantly, movement-based tasks can be adjusted to explore and compare the neural underpinnings of both cooperative and competitive interactions without altering the task nature. For instance, in the study conducted by Liu et al. (2021), a tennis-like sports game equipped with motion sensors was employed to investigate both cooperation and competition by asking individuals to play together against two computerized opponents or to play one against the other. Similarly, movement-based paradigms can be used to study and compare simultaneous and turn-based tasks, as seen in Naeem et al. (2012), who asked individuals to synchronize their respective finger tapping in either in-phase (concurrent) or anti-phase (turn-based) mode.

It is also worth mentioning that movement-based tasks have been used so far to investigate only similar joint actions: no study has implemented a movement-based paradigm to investigate complementary joint actions. For instance, a real-world complementary joint action task based on movement could involve one individual pouring water from a bottle into a glass held by another individual. A similar task was used by (Ménoret et al. 2014) , who implemented an EEG-hyperscanning setup and paradigm to study non-verbal joint action but limiting the analysis to a within-brain level.

#### Music-based tasks

Music-based tasks are ecologically valid scenarios to study cooperation and interpersonal interactions in general (Keller et al. 2014, Acquadro et al. 2016, Novembre and Keller 2018) and have been extensively used in real-world joint action research. These tasks required individuals to play music together, primarily using guitar (Lindenberger et al. 2009, Sänger et al. 2012, 2013, Müller et al. 2013, 2018, Müller and Lindenberger 2019, 2022, 2023) or piano (Zamm et al. 2021, Gugnowska et al. 2022).

Music-based experimental tasks offer several key advantages:

1. High experimental control over the variables studied in real-world contexts.
2. Ability to easily investigate INC not only between two individuals but also among three (Ramírez-Moreno et al. 2022) or four (Müller et al. 2018, Müller and Lindenberger 2023) individuals simultaneously, without significantly increasing methodological complexity.
3. Absence of full-body movement artefacts, thus reduces technical complexity and data processing requirements.
4. Flexibility in studying both similar and complementary joint actions. For instance, researchers can ask musicians to play the same instrument either in the same tune (similar joint action; Müller and Lindenberger 2023) or in different voices (complementary joint action; Gugnowska et al. 2022), or to play different instruments (complementary joint action; Ramírez-Moreno et al. 2022).

However, music-based tasks present the disadvantage of being unsuitable to investigate competition.

#### Computer-based tasks

Together with movement-based tasks, computer-based tasks are the most employed ones in EEG-hyperscanning studies of joint action, underscoring the adaptability of technology for joint action research purposes. A common computer-based task requires two individuals playing a videogame. Unlike music-based tasks and, more easily than movement-based ones, computer gaming tasks facilitate the study and comparison of cooperative and competitive joint actions: three out of four studies focusing on competition opted for this type of task (Sinha et al. 2016, Léné et al. 2021, Susnoschi Luca et al. 2021). This preference can be attributed to the ease of implementation when compared to movement-based tasks. In fact, competitive tasks may necessitate the creation of scenarios that induce rivalry, conflict, or the pursuit of individual goals, which can be challenging to design in a real-world context without introducing unwanted variables or biases. Conversely, in a computer gaming paradigm individuals can engage in competitive joint actions by simply modifying the game rules or objectives, thereby creating a controlled and easily adjustable environment for investigating competitive interactions (Léné et al. 2021, Susnoschi Luca et al. 2021).

Furthermore, computer gaming paradigms could also facilitate the study and comparison of simultaneous and turn-based joint actions. For instance, the computer-based task implemented by Strang et al. (2015) involved two individuals simultaneously controlling a spaceship in a simulated ‘asteroid field’ using a handheld gamepad. On the other hand, the computer-based task implemented by Sinha et al. (2016) involved two individuals playing a computerized pong-game, in which they virtually hit the ball in an alternating sequence. Nevertheless, to date, no study has directly compared simultaneous and turn-based joint action within the same computer-based paradigm.

#### Piloting simulation paradigms

EEG-hyperscanning paradigms simulating vehicle piloting have been less popular than other non-verbal joint action paradigms. However, they are particularly suitable to investigate cooperative, simultaneous, and complementary joint actions (Astolfi et al. 2011, 2012, Toppi et al. 2016, Dehais et al. 2021) and offer a distinctive perspective on coordination, teamwork, and information exchange during intricate tasks. In fact, unlike simpler motor tasks, simulated piloting tasks encompass both cognitive and sensory-motor components, enabling researchers to delve deeper into the neural mechanisms that underpin coordinated actions in scenarios closely resembling real-world teamwork situations.

Furthermore, paradigms based on piloting simulation typically involve well-defined and distinct roles and actions that individuals must perform together to achieve a common goal. For instance, individuals interacted to pilot and co-pilot an aircraft. These roles involve specific and different actions and responsibilities, creating a clear division of tasks within the joint action (Astolfi et al. 2011, 2012, Toppi et al. 2016, Dehais et al. 2021). Therefore, paradigms based on piloting simulation can contribute to expanding our knowledge on joint actions by elucidating the complexities of cooperative endeavours in demanding, high-stakes contexts, which might be less evident in simpler experimental setups.

### INC measures and key findings

The first data analysis approach used to measure INC was based on phase synchrony (Lindenberger et al. 2009), which includes various metrics, the most popular being Phase Locking Value (PLV; Léné et al. 2021, Liu et al. 2021, Maÿe et al. 2021, Susnoschi Luca et al. 2021, Gugnowska et al. 2022). Phase synchrony metrics are employed for their ability to assess the alignment of the neural activity of interacting individuals in time, making these metrics particularly suitable for scenarios where precise timing of actions is crucial for task success. Consequently, they have been extensively used in cooperative joint actions such as music-based tasks, where all interacting individuals share the common goal of precisely coordinating their movements temporally to achieve synchronous outcomes. In such cases, researchers have observed INC among the brains of musicians involved in joint action, primarily in fronto-central areas in the delta, theta, and alpha bands (Lindenberger et al. 2009, Sänger et al. 2012, Müller et al. 2013, Gugnowska et al. 2022, Müller and Lindenberger 2022).

However, due to their strong emphasis on timing, phase synchrony metrics are not ideal when studying INC in tasks characterized by substantial variability in the frequency of individuals’ actions, as in the case of competitive joint actions, where variability is fundamental to the motor strategy. In fact, in competition individuals coordinate their movements to support joint action while aiming to disrupt this coordination privately for their advantage, aiming to win. As a result, individuals’ movements become highly unpredictable and variable. Therefore, the timing of actions can exhibit considerable fluctuations, posing challenges for precise measurement, as also indicated by Léné et al. (2021). This may explain why two out of three studies using phase synchrony metrics to investigate INC in competitive tasks did not find significant results (Léné et al. 2021, Liu et al. 2021).

In scenarios where the timing of actions exhibits significant variability—encompassing not only competitive joint actions but also those characterized by turn-based interactive dynamics—metrics based on amplitude/envelope correlation were preferred to capture the strength of INC. Notably, researchers such as Liu et al. (2021) and Sinha et al. (2016) have effectively used the Pearson correlation coefficient to compare competition and cooperation. Additionally, Kawasaki et al. (2018) employed the cross-correlation measure in a cooperative and turn-based task, reporting the presence of INC between the interacting individuals.

Coherence is another commonly used metric in EEG-hyperscanning studies to assess INC during joint actions, both in cooperation and competition. Coherence quantifies the correlation between two signals over a specific time window as a function of frequency. In terms of INC, coherence quantifies how the EEG activity of two individuals relates in terms of phase and amplitude at a certain frequency. This makes coherence particularly useful for examining INC in scenarios where both phase and amplitude of EEG activity are relevant for the success of the joint action. An example is the dyadic juggling task used by Filho et al. (2016), where jugglers had to coordinate not only the timing of their participatory actions (which could be associated with a metric of phase synchrony) but also the intensity of their actions (which could be associated with a measure of amplitude correlation). In this study, the authors used coherence to quantify INC and found inter-brain coupling involving frontal, central, parietal, and occipital cortical regions in the theta and alpha frequency bands.

In general, coherence metrics, such as the wavelet transform coherence, have been used to measure INC primarily in the category of simultaneous and cooperative joint actions. These studies have consistently reported increased INC of theta and alpha bands in frontal, central, and parietal brain regions (Léné et al. 2021, Ogawa and Shimada 2023, Balconi and Angioletti 2023a, 2023b). Notably, Léné et al. (2021) extended this approach to investigate differences between cooperation and competition. Their findings suggested that frontal alpha and parietal theta are more prominently involved in cooperation, whereas competition entails increased activation of alpha, along with parietal and occipital theta. However, when considering complementary joint actions, coherence may not be the best method for capturing the dynamics of INC. In fact, in complementary joint actions, interacting individuals have distinct roles and perform different actions that may require the activation of different brain areas at various frequencies. Therefore, the nature of this type of joint action makes it challenging to detect meaningful results using coherence metrics. This limitation becomes evident in the study of Maÿe et al. (2021), where they explored INC during a complementary computer gaming task without finding significant results.

Conversely, causality/directionality measures have been particularly used to study INC in complementary joint action. Diverging from other methods that rely on symmetric and non-directional INC measures, this approach delves into the causal relationship and direction of information flow across the activated brain areas, and has been extensively employed especially to investigate leader-follower dynamics within tasks featuring predefined or task-determined roles, such as in piloting simulation studies (Astolfi et al. 2011, 2012, Toppi et al. 2016). In these investigations, researchers employed Partial Directed Coherence (PDC) and identified a distinct engagement of frontal and parietal theta and alpha bands, exhibiting a unique pattern of directionality from the leader to the follower, and the vice versa.

Notably, studies utilizing PDC in computer-based tasks failed to discern any causality/directionality (Astolfi et al. 2014, 2020, Maÿe et al. 2021), maybe because in such context the actions of one individual do not directly influence the behaviour of the other; instead, reciprocal influence at the behavioural level is mediated by a computer, shaping the action and interaction environment. This observation underscores the notion that the causality/directionality approach may be better suited for investigating INC in complementary joint actions, but where roles and directions of influence are clearly defined, such as in tasks like piloting simulation and music-based activities (Müller et al. 2013, 2016, Müller and Lindenberger 2019). These contexts, characterized by well-defined roles, enhance the robust identification of causality and directionality in INC. By employing PDC in these studies, researchers could identify a specific engagement of the frontal and parietal theta and alpha bands, manifesting distinct patterns of directionality from the leader to the follower, and the vice versa.

Building on the diverse approaches used in EEG-hyperscanning studies, particularly phase synchrony, coherence, and causality/directionality, the graph theory approach has emerged as the most popular method. However, its application has been limited to the study of cooperative and simultaneous joint actions where individuals perform either similar or complementary activities. Differently from other methods that focus on identifying whether and where INC occurs among interacting individuals, graph theory measures are used to study how brains potentially organize and structure their activity during joint action. The main advantage offered by the graph theory approach is the possibility of characterizing the organization of neural activations. In this context, several measures can provide unique insights into the organization and structure of the functional brain networks and dynamics during joint actions.

A commonly used graph metric is strength, which reflects the robustness of interconnections between two or more brains or specific brain regions during joint actions. Research findings suggest that when two or more individuals engage in a joint action, the strength of INC between their brains increases. Examples include heightened strength during a romantic kiss compared to kissing one’s own hand (Müller and Lindenberger 2014), in a musical quartet versus the audience during a musical performance (Müller and Lindenberger 2023), and during periods of music performance compared to non-music periods (Sanger et al. (2012). In the specific domain of music-based tasks, the intensity of interconnections between brains was notably higher at lower frequencies (delta and theta), with a more pronounced involvement of the frontal areas (Müller et al. 2013, 2018). Other findings suggest that the strength of INC varies based on the roles of leader and follower (Sänger et al. 2012, 2013).

Another significant graph metric is density, which quantifies the ratio of existing connections to the total possible connections. This metric introduces an additional layer to our understanding of network organization and coordination dynamics, providing insights into how densely interconnected the brains or specific brain regions are during joint actions. Research findings suggest that density increases during the performance of joint actions compared to non-interactive conditions or where interaction is with a computer (Astolfi et al. 2014, 2020, Toppi et al. 2015, 2016).

Characteristic path length (CPL) and clustering coefficient (CC) have also been extensively used to investigate INC during joint actions. These graph metrics play a fundamental role in examining how the brain activity of two interacting individuals organize to support the shared motor task, elucidating the strategies of network integration and segregation. CPL represents the average shortest path between all pairs of nodes in the network: low values of CPL indicate integration between brains during joint action. Conversely, CC assesses the degree to which nodes in a network tend to cluster together. Hagh values of CC indicate a segregated network, where regions within the brain preferentially connect with nearby regions. Research findings suggest that INC networks tend to be more integrated (i.e. lower values of CPL associated with higher values of CC) in similar joint actions (Müller et al. 2013, Müller and Lindenberger 2014, 2023, Astolfi et al. 2020) as compared to complementary joint actions (Sänger et al. 2012, Müller et al. 2018). Similar patterns emerge when considering global (GE) and local efficiency (LE). GE represents how effectively information is transferred across the network, whereas LE assesses the effectiveness of information exchange within local neighbourhoods in the network. Research indicates that LE increases in similar (Astolfi et al. 2020, Müller and Lindenberger 2023) and complementary joint actions (Müller et al. 2018), whereas GE consistently shows higher values in similar joint actions (Toppi et al. 2015, Astolfi et al. 2020, Müller and Lindenberger 2023). However, in the case of complementary joint actions, results are inconsistent, with Dehais et al. (2021) reporting an increase of GE and Müller et al. (2018) reporting a decrease of GE.

In addition to these six approaches, other methodologies have been employed to investigate INC from various perspectives. Mutual Information is a metric of signal entropy that explores the amount of shared information between the neural activities of interacting individuals. Results obtained with this approach indicate that, more information is shared between the brains of interacting individuals during turn-based as compared to simultaneous joint actions (Naeem et al. 2012). Therefore, Mutual Information could help elucidating the dynamics of information sharing during different types of joint actions. Bispectrum assesses the non-linear interactions between brains by quantifying the degree of coupling between different frequency components of the neural signals, thus offering a unique perspective on the complexity of joint actions. Using this method, Ramírez-Moreno et al. (2022) found that INC increased when more musicians were performing together and during synchronized performance as compared to desynchronized performance. These results underscore the ability of Mutual Information to capture INC within the context of joint actions.

In most studies, data analysis has revealed the occurrence of INC during joint action, although with different patterns related to the specific features of the joint action tasks. However, it is worth noting that the theta, alpha, and beta frequency bands were primarily investigated, with little attention devoted to the delta and gamma bands. Similarly, cortical areas such as the frontal, central, and parietal regions have been more frequently associated with INC, whereas the occipital and temporal areas have been less reported. Several factors may contribute to these trends and research gaps. First, some studies selectively focused on specific frequency bands and cortical areas, potentially introducing a representational bias. For instance, some studies might have deliberately focused on the theta, alpha, and beta bands and on the frontal, central, and parietal areas due to their established roles in motor, cognitive and social processing (e.g. Filho et al. 2016). We should also keep in mind that these frequency bands and cortical areas might have been selected to avoid the interference of artefacts related to movements or myogenic activity in the EEG recordings, mainly in the delta (Mihajlović et al. 2014, Gorjan et al. 2022) and gamma frequency bands (Muthukumaraswamy 2013, Liebisch et al. 2021), respectively. Traditional methods like filtering (or adaptive filtering) and blind source separation (BSS) (e.g. Independent Component Analysis—ICA) are underperforming or even ineffective in removing movement-related interference because of its complex and unstructured nature, unlike most physiological artefacts such as eyeblinks or eye movements. So far, the only method specifically developed for removing transient high amplitude artefacts from EEG recordings is Artifact Subspace Reconstruction (ASR) (Chang et al. 2020). However, it needs motion-free EEG segments as reference and may generate high frequency noises, and no study included in this review has employed it.

### Limitations of the study

Despite the numerous advantages of using a systematic scoping review to explore the literature on INC during joint actions, the present work has some limitations that warrant attention. First, we focused on studies published in English languages and from specific databases, potentially excluding valuable research from other languages or unpublished sources, therefore introducing a possible publication bias. Secondly, the choice of focusing on EEG-hyperscanning studies may limit the generalizability of the findings to other methods. Furthermore, the diversity in research methodologies, paradigms, and INC measures made direct comparisons challenging. Furthermore, many studies involved small samples, mostly young adults, which limit the applicability of the findings to broader or more diverse populations.

Finally, an inherent limitation of the scoping review methodology is the lack of a systematic assessment of the methodological quality of the included studies. While a scoping review is particularly useful for mapping a broad field of research and identifying gaps in the literature, it does not provide an in-depth evaluation of the methodological rigour of the studies considered, nor does it offer a critical analysis of potential biases or research errors.

## Conclusions

In recent years, the neuroscientific study of joint action has experienced significant growth and development, with much attention devoted to studying cooperative, simultaneous, and similar joint actions, whereas competitive, turn-based, and complementary joint actions have received less attention. Most studies have focused on dyadic joint actions due to their versatility and the practical advantages offered in terms of experimental control and data analysis. The study of joint action has been enriched by a variety of tasks, each offering distinctive insights and challenges, collectively enhancing our understanding of these phenomena. Notably, achieving a balance between experimental control and ecological validity remains an ongoing challenge in joint action research (Kasai et al. 2015, Matusz et al. 2019, Shamay-Tsoory and Mendelsohn 2019, Hamilton 2021).

Diverse analytical approaches have been employed to investigate the neural underpinnings of joint action, each shedding light on different aspects of INC (Zamm et al. 2023a). Research results have consistently revealed the occurrence of INC during joint action, although with variations depending on the specific tasks. Primary findings regarded the theta, alpha, and beta frequency bands, and the frontal, central, and parietal cortical areas, likely due to their established roles in motor, cognitive, and social processing. However, it is important to consider that other frequency bands (i.e. delta and gamma) and cortical areas (i.e. temporal and occipital), more prone to artefacts and noise contamination, have been neglected during the analysis to avoid potential bias and confounding factors. Addressing these limitations is crucial for a more comprehensive understanding of INC.

In conclusion, the present systematic scoping review mapped EEG-hyperscanning studies on non-verbal joint action. It relied on a task classification system that permits to categorize the main findings in relation to the joint action type, which is an important aspect to consider because in real-life experience a variety of joint actions occur that are sustained by different motor and neural strategies. Utilizing such a classification system helped us to better systematize the information contained in the literature and to identify trends and gaps in this research field. This systematic scoping review also considered the type of method used for data processing, highlighting the close relationship between the task and the analytical approach to be employed to investigate INC in joint actions. Future studies should consider this close relationship when choosing the task and data analysis approach, but also in the formulation of the work hypotheses and in the discussion of the results obtained. This review could serve as a roadmap for those navigating the field of neuroscientific research on joint action, offering an overview of the tasks used, approaches utilized, and their relationship in each category of joint action in EEG-hyperscanning studies.

To advance the understanding of INC during joint actions, future investigations should expand beyond the conventional dyadic designs and incorporate more diverse group dynamics, including larger teams, which are more representative of real-world scenarios. Moreover, integrating a broader range of joint action types, such as competitive, complementary, and turn-based actions, into the EEG-hyperscanning paradigm could provide novel insights. While the current literature has primarily focused on the frontal, central, and parietal brain areas, future studies should explore the role of less-studied brain regions, such as the temporal and occipital areas. Another avenue for further research lies in examining the role of individual differences, including age, expertise, and personality, in shaping the neural dynamics of joint action. Aspects such as empathy, social context, and task difficulty level could also be considered to understand how these factors influence the effectiveness and nature of INC.

Lastly, advancements in analytical techniques, such as novel methods to remove movement-related artefacts for joint action paradigms employing full-body movements, or machine learning algorithms or multi-modal neuroimaging approaches for a deeper understanding of inter-brain dynamics, could provide a more nuanced understanding of the causal relationships and temporal dynamics of INC in joint actions. Incorporating more ecologically valid tasks that resemble real-world interactions could help improving the external validity of future findings and facilitating the translation of research to practical applications in fields such as education, therapy, and team performance optimization.

## Supplementary Material

nsaf050_Supplementary_Data
