# Supplementary material for: Non-verbal joint action in healthy adults: a systematic scoping review of EEG-hyperscanning research
Source: Soc Cogn Affect Neurosci. 2025 May 8;20(1):nsaf050. doi: 10.1093/scan/nsaf050 (PMC12393900; doi:10.1093/scan/nsaf050)
Supplement: nsaf050_Supplementary_Data [file nsaf050_supplementary_data.zip › scan-24-254-File010.docx]

**Appendix B**

1. **METHODS**

The current systematic scoping review was designed following the guidelines of Arksey and O’Malley (2005) and Levac et al. (2010), and it was prepared according to the checklist of PRISMA-ScR (Preferred Reporting Items for Systematic Reviews and Meta-Analyses Protocols Extension for Scoping Reviews; Tricco et al., 2018; see Appendix A).

In particular, the current systematic scoping review is characterized by the following five mandatory steps:.

***STEP 1 - Research questions and operational definition of joint action***

The research questions of the present systematic scoping review are:

1. *What are the joint action tasks used in EEG-hyperscanning studies?*
2. *What are the analytical approaches used to study INC?*
3. *What are the key findings associated with INC?*

To answer these research questions, an operational definition of joint action is needed. To date, no generally accepted definition is existing. Therefore, we introduce a new operational definition of joint action based on the theoretical framework developed by Clark (1996) in his studies on verbal joint actions in the research on psycholinguistics. In particular, Clark identified seven basic principles of joint action, two of which can be used to conceptually define joint action: the participant principle and the public goal principle. The participant principle asserts that joint actions involve at least two individuals. While important, this principle alone cannot differentiate joint action from other interpersonal interactions. The specificity of joint action lies in the shared public goal principle, where interacting individuals have the intention to achieve a shared and public goal. This goal is recognized by all participants and cannot be attained through individual actions alone. It is this public shared goal that binds participants together in a mutually interdependent manner. Clark emphasized the notion of participatory (i.e., mutual interdependent) actions, distinguishing it from the notion of autonomous (i.e., independent) actions.

Therefore, building on Clark (1996)'s theoretical framework, we define joint action as:

“Any form of interpersonal interaction whereby two or more individuals coordinate their participatory actions in space and time to achieve a shared and public goal.”

Notably, this definition is also aligned with the well-known concept of mutual joint control interaction in social psychology (Kelley et al., 2003) and with the concept of interdependent interpersonal interaction introduced social neuroscience (Liu and Pelowki, 2014). Additionally, it is consistent with other conceptualizations of joint action proposed in other research fields, including philosophy of action (Searle et al., 1990; Bratman, 1992), social psychology (Deutsch, 1949), and cognitive psychology (Vesper et al., 2010). By emphasizing the interdependence of participants and the pursuit of a shared public goal, this definition captures the essence of joint action and facilitates the review of existing literature.

***STEP 2 - Search strategy***

Multiple bibliographic databases of different scientific disciplines were used to select the studies of interest for the present review. In particular, IEEE Xplore was used to cover the engineering and technology literature; PubMed was used to provide a health science point of view; Web of Science and Scopus were used for its cross-disciplinary perspective; and the Directory of Open Access Journals (DOAJ) was used to include all open access journals. To uncover any additional publications or grey literature, reference lists, Google Scholar, and academic social networks (i.e., Research Gate) were included.

A focused iterative selection strategy was adopted for the systematic scoping review. In particular, the search first focused on studies using a hyperscanning approach to investigate the brain-to-brain dynamics of interpersonal interactions. To this aim, one of the authors listed the keywords that appeared on hyperscanning publications. Then, two authors selected the keywords considered most relevant for the purpose of the systematic scoping review. Initial keywords used were: “hyperscanning”, “hyperbrain”, “social cognition”, “joint action”, “EEG”, “MEG”, “fMRI”, “fNIRS”, “social interactions”. The keywords “social cognition”, “joint action”, and “EEG” were highly relevant but too broad to be included. Keywords as “MEG”, “fMRI” and “fNIRS” were outside the current scoping purposes. Disagreements about the inclusion terms were solved through discussion and consensus. At the end of this process, two independent search terms were retained: “hyperbrain” OR “hyperscanning”, which permitted to include all potentially relevant studies (e.g., hyperscanning studies performed with different brain monitoring techniques). The selection of the relevant studies was performed in the subsequent steps of the funnel search.

***STEP 3 - Study selection***

Only hyperscanning studies of joint action performed with EEG and published until August 31st, 2023 were selected by means of a careful screening of the abstracts and full texts. As specified at the beginning of the Methods section, quality metrics of the selected studies were not assessed because it was deemed not relevant for the scoping review methodology.

Original studies were included if they satisfied the following conditions: (1) they were either full original articles, peer-reviewed conference papers or original book chapters; (2) they described hyperscanning studies; (3) brain activity was monitored using EEG; (4) the articles included an experimental design; (5) a joint action task coherent with our novel operational definition was employed (see section 2.1); (6) the joint action employed was performed without any verbal communication; (7) the article included analyses and results related to INC; (8) articles were written in English. Exclusion criteria were: (1) the studies were described only as conference abstracts; (2) articles did not include an experimental design; (3) a technique different from EEG-hyperscanning was used; (4) the study did not include results related to INC; (5) the study was performed on non-human subjects; (6) children or elders were recruited for the study (i.e., <18 and > 60 years of age); (7) non-healthy participants were recruited for the study; (8) the experimental design included the use of verbal communication and/or an interpersonal interaction task that did not comply with the operational definition of joint action; (9) articles written in a language different from English. In case of doubt, a third author was consulted.

The web version of EndNote (Clarivate Analytics) was used as a reference management software to collect and list the titles and abstracts derived from the article search. Once duplicates were removed, six categories were created according to the inclusion and exclusion criteria, each containing:

1. articles included because related to EEG-hyperscanning experiments;
2. articles excluded because related to hyperscanning experiments that used techniques different from EEG;
3. articles excluded because they did not include an experimental design (i.e., non-hyperscanning studies);
4. articles excluded because they related to studies involving non-humans;
5. articles excluded because they are not accessible online;
6. articles excluded because they are written in a language other than English.

Subsequently, category 1, containing the articles related to EEG-hyperscanning experiments, was divided in four sub-categories:

1. articles considered for the present systematic scoping review (i.e., included articles);
2. articles excluded because the experimental task did not satisfy the operational definition of joint action and/or verbal communications were included;
3. articles excluded because the study population included children, elders, and/or non-healthy subjects.
4. articles excluded because they are not reporting INC-related results.

The remaining articles were further analyzed as relevant for the present systematic scoping review.

For sub-groups a and b, the two authors engaged in a thorough discussion to ensure that the studies considered for this review indeed employed tasks that were fully aligned with our operational definition of joint action. Additionally, the two authors carefully reviewed the studies of sub-group b to confirm that no study was erroneously excluded because of a false evaluation of the task that was, in fact, consistent with our operational definition of joint action. This collaborative process facilitated accurate classification and consensus regarding the inclusion and exclusion of studies in the present review.

***STEP 4 –Extraction of the relevant information***

The proposed operational definition of joint action provides a comprehensive and unambiguous framework for distinguishing joint action from other forms of interpersonal interaction. Nevertheless, it is important to acknowledge the existence of diverse manifestations of joint action.

Therefore, for a precise study of joint action, a definition is mandatory but not sufficient: a classification system that delineates and categorizes the various types of joint action based on fundamental task characteristics is imperative. Such an approach has been previously implemented for general interpersonal interactions in the fields of social psychology (Kelley et al., 2003) and social neuroscience (Liu and Pelowski, 2014), respectively.

To extend this approach within the specific yet broad domain of joint action, we propose a new classification system in accordance with the theoretical framework of Clark (1996). Specifically, we employed three basic principles identified by Clark in his theoretical framework, which can be used as key properties for the sub-categories of joint action:

1. Private goals principle: Individuals engaged in joint actions have both shared public goals and personal interests. When these private interests align, the joint action is cooperative: the achievement of one individual's private interest facilitates the achievement of the private interests of the other individual(s) involved. Conversely, when the individuals’ private interests are in conflict, the joint action becomes competitive: achieving one individual's private interest obstructs the achievement of the private interest(s) of other individual(s). Notably, private interests always subordinate to the shared goal required for joint action. This principle aligns with the concept of covariation of interests (Kelley et al., 2003) and goal structure (Liu and Pelowski, 2014) used in previous classification systems, and is consistent with earlier theories in social psychology (Kelley, 1991; Deutsch, 1949).
2. Dynamics principle: Any joint action is characterized by a specific temporal structure that describes how the individuals interact with each other and how they coordinate their participatory actions. Based on this principle, joint actions are categorized as: simultaneous, when the interacting individuals perform their participatory actions concurrently (e.g., dancing a pair dance); or turn-based, when the interacting individuals coordinate their actions in a turn-taking style with a given timing between the actions of the different individuals (e.g., playing tennis). The dynamics principle is similar to the key property that Kelley et al. (2003) called 'response condition' and to the interactive structure defined by Liu and Pelowski (2014).
3. Activity role principle: Individuals engaged in joint action can either perform equal activities, e.g., when two individuals dance a tango or play tennis, or engage in different but complementary activities to achieve the shared goal, e.g., an individual pours water from a bottle into a glass held by another individual. Remarkably, the activity role principle aligns with the key property of 'similarity of individuals' actions', which is employed to resolve coordination problems, as described by Kelley et al. (2003). For the current work, we will refer to this characteristic of the task as activity similarity.

Our proposed classification system of joint action is graphically presented in **Figure 1**.


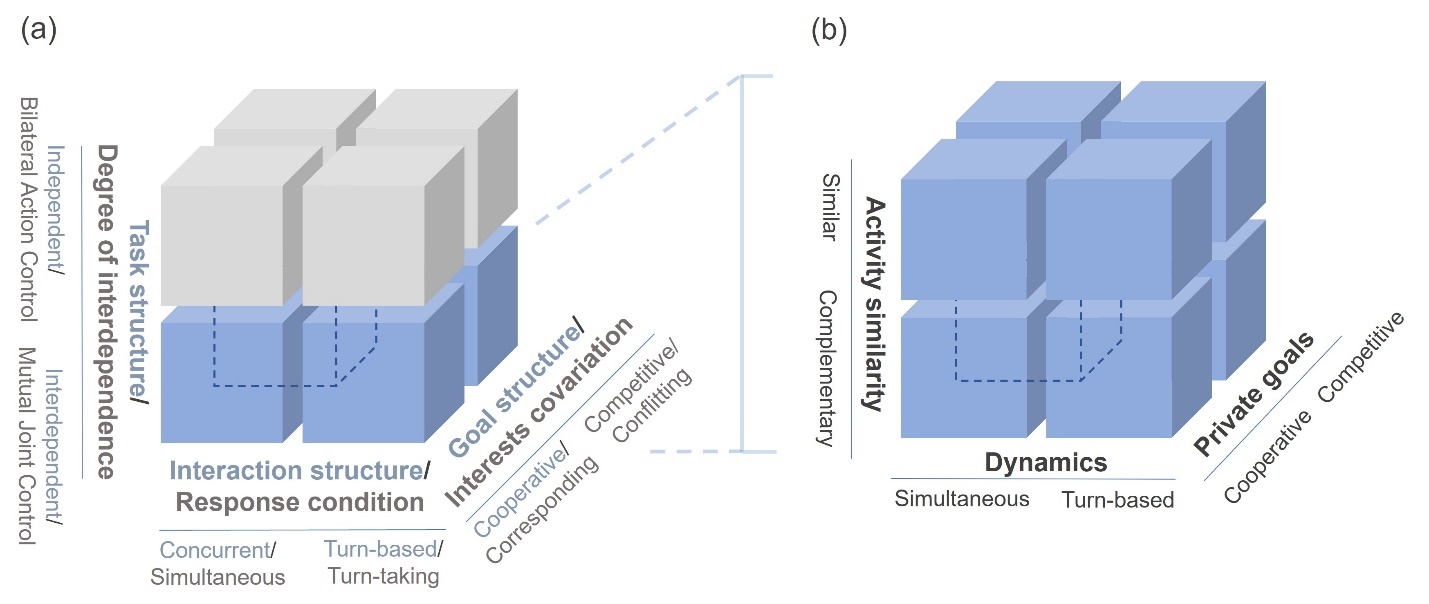
**Figure 1.** Categorization of the non-verbal joint action: (a) graphical representation of the similarities between the categorization model of interpersonal interactions proposed by Liu & Pelowski (2014b) (text in blue) and characteristics of the task used by Kelley et al. (2003) in their Atlas of Interpersonal Situation (text in gray). Gray cubes refer to independent interpersonal interactions, while blue cubes refer to interdependent interpersonal interactions. (b) Graphic representation of the categorization model of non-verbal joint actions based on the characteristics of the task proposed in the present systematic scoping review. Dashed lines indicate that joint action is a specific sub-category of interpersonal interactions, which corresponds to interdependent interaction (blue cubes).

After having categorized the selected studies, we extracted the following information:

1. publication year, authors, title, and article type (i.e., full original article, extended conference paper, or original book chapter);
2. study objectives;
3. specific type of joint action task used, including the public and shared goal of the joint action task employed, the number of interacting individuals and how the task was implemented;
4. the metrics employed to quantify INC, including methods used to pre-process EEG data after filtering, if any;
5. the related main findings in EEG patterns and cortical areas involved in joint action.

Regarding the main findings, it is worth noting that our data collection approach was designed to provide a concise and non-interpretative summary of the INC phenomena. By using the developed data-extraction procedure, one author extracted data from five randomly selected studies and determined whether the adopted data extraction approach was consistent with the research questions.

This data extraction procedure allowed us to compile a rich dataset that served as the basis for our systematic analysis. The extracted information was instrumental in fulfilling our objectives.

***STEP 5 - Results reporting***

The study results will be presented in a structured manner, aligning with the research questions outlined earlier. We will present the gathered information, that regards: (1) the joint action categories investigated, distinguishing between cooperative and competitive, simultaneous and turn-based, similar and complementary joint actions, and between dyadic, triadic, and group-based (> 3 interacting individuals) joint actions; (2) the joint action tasks that have been employed to implement the respective joint action categories; (3) the metrics employed to quantify INC, distinguishing between (I) phase synchrony-based, (II) amplitude/envelope correlation-based, (III) coherence-based, (IV) causality-based, (V) graph theory-based, and (VI) other approaches; (4) the main findings related to INC, focusing on the main frequency bands and cortical areas reported to be involved within each category and joint action task.

All information will be presented in tabular and descriptive formats. Graphical representations will be utilized whenever possible to provide an immediate macroscopic visual overview of the main review findings.
